# Access to principal treatment centres and survival rates for children and young people with cancer in Yorkshire, UK

**DOI:** 10.1186/s12885-017-3160-5

**Published:** 2017-03-04

**Authors:** Lesley Fairley, Daniel P. Stark, Daniel Yeomanson, Sally E. Kinsey, Adam W. Glaser, Susan V. Picton, Linda Evans, Richard G. Feltbower

**Affiliations:** 1 Division of Epidemiology and Biostatistics, School of Medicine, Worsley Building, University of Leeds, Clarendon Way, Leeds, UK LS2 9JT; 2 St James’s Institute of Oncology, Leeds Institute of Cancer and Pathology, University of Leeds and Leeds Teaching Hospitals NHS Trust, Bexley Wing, St James’s Hospital, Beckett Street, Leeds, LS9 7TF UK; 3 Paediatric Oncology and Haematology Department, Sheffield Children’s Hospital, Western Bank, Sheffield, S10 2TH UK; 4 Leeds Teaching Hospitals NHS Trust, Leeds General Infirmary, Great George Street, Leeds, LS1 3EX UK; 5 Sheffield Teaching Hospitals NHS Foundation Trust, Weston Park Hospital, Whitham Road, Sheffield, S10 2SJ UK

**Keywords:** Specialisation, Children, Teenager and young adult, Survival, Principal Treatment Centres

## Abstract

**Background:**

Principal Treatment Centres (PTC) were established to provide age-appropriate care as well as clinical expertise for children and young people with cancer. However, little is known about the effects of specialist treatment centres on survival outcomes especially for teenagers and young adults. This population-based study aimed to describe access to PTC and the associated trends in survival for 0–24 year olds accounting for stage of disease at presentation and treatment.

**Methods:**

Patients diagnosed from 1998–2009 aged 0–24 years were extracted from the Yorkshire Specialist Register of Cancer in Children and Young People, including information on all treating hospitals, followed-up until 31st December 2014. The six commonest cancer types were included: leukaemia (*n* = 684), lymphoma (*n* = 558), CNS tumours (*n* = 547), germ cell tumours (*n* = 364), soft tissue sarcomas (*n* = 171) and bone tumours (*n* = 163). Treatment was categorised into three groups: ‘all’, ‘some’ or ‘no’ treatment received at a PTC. Treatment at PTC was examined by diagnostic group and patient characteristics. Overall survival was modelled using Cox regression adjusting for case-mix including stage, treatment and other socio-demographic and clinical characteristics.

**Results:**

Overall 72% of patients received all their treatment at PTC whilst 13% had no treatment at PTC. This differed by diagnostic group and age at diagnosis. Leukaemia patients who received no treatment at PTC had an increased risk of death which was partially explained by differences in patient case-mix (adjusted Hazard Ratio (HR) = 1.73 (95%CI 0.98–3.04)). Soft tissue sarcoma patients who had some or no treatment at PTC had better survival outcomes, which remained after adjustment for patient case-mix (adjusted HR = 0.48 (95%CI 0.23–0.99)). There were no significant differences in outcomes for other diagnostic groups (lymphoma, CNS tumours, bone tumours and germ cell tumours). For leukaemia patients survival outcomes for low risk patients receiving no treatment at PTC were similar to high risk patients who received all treatment at PTC, implying a benefit for care at the PTC.

**Conclusion:**

This study demonstrates that for leukaemia patients receiving treatment at a PTC is associated with improved survival that may compensate for a poorer prognosis presentation. However, further information on risk factors is needed for all diagnostic groups in order to fully account for differences in patient case-mix.

**Electronic supplementary material:**

The online version of this article (doi:10.1186/s12885-017-3160-5) contains supplementary material, which is available to authorized users.

## Background

Cancers in children and young people are rare, accounting for approximately 1% of all cancers diagnosed in the UK, however, cancer is one of the leading causes of death in this age group [1]. The National Institute for Health and Clinical Excellence Improving Outcomes Guidance (IOG) for cancer in children and young people published in 2005 aimed to improve outcomes for this population by recommending the provision of age-appropriate care and clinical expertise [2]. Principal Treatment Centres (PTC) were established, which operate within high volume cancer centres to provide specialist care for this group of patients. There are currently 21 paediatric PTCs in the UK and Ireland where the majority of children, aged 15 and under, with cancer are treated [3]. For paediatric cancers there is evidence that higher volume hospitals and specialist hospitals provide care with better outcomes, however many studies included in a recent systematic review were unable to account for stage of disease and other confounding factors such as age, sex, treatment and other patient demographics [4].

Services for teenagers and young adults (TYA) have developed since 2005 and there are currently 25 designated TYA PTC in England [5, 6]. Some PTC include units for both children and TYAs. Cancer patients aged 16 to 18 years are managed at a PTC and those aged 19 to 24 years are assessed at a PTC and given an informed choice where they are treated [2]. Analysis of TYA hospital admissions (aged 15–24) in England between 2001 and 2006 found that many TYA patients received little or no inpatient treatment at TYA specialist centres [7]. However, little is known about the effects of specialist treatment centres on survival for TYA.

The aims of this paper were to describe the patterns of care for the population of children and young people with cancer in Yorkshire between 1998 and 2009, therefore including periods before and after t﻿he establishment of PTCs for TYA, and assess the association with trends in survival, accounting for clinical prognostic factors which we are unique in being able to assess, notably stage and treatment using data from a specialist cancer register.

## Methods

### Study population

Data were extracted from the Yorkshire Specialist Register of Cancer in Children and Young People (YSRCCYP), which is a population based database of children and young people (0–29 years) diagnosed with cancer residing in the Yorkshire and Humber region in England. The primary source of ascertainment was hospital records via the PTC with secondary sources including neuropathology reports, hospital admissions and other regional and national cancer registries [8]. The database contains detailed information on clinical prognostic factors including detailed treatment and stage information.

Data on all registered neoplasms diagnosed in children (0–14 years) and TYA (15–24 years) between 1998 and 2009 were extracted. Diagnoses were categorised into histological groups according to the International Classification of Childhood Cancer 3 (ICCC-3) [9]. We included the six most common main diagnostic groups in this age range: leukaemia, lymphoma, central nervous system (CNS), malignant bone tumours, soft tissue sarcomas and germ cell tumours corresponding to ICCC codes I, II, III and VIII, IX and X, respectively. Biennial proactive follow-up of cases was carried out to ascertain each individual’s vital status, with a censoring date of 31st December 2014.

For each patient we identified all hospitals where they received any treatment (surgery, radiotherapy or chemotherapy). Each hospital (whether in Yorkshire or outside) was classified into one of two groups: PTC or non-PTC based on designation for NHS quality reviews. Hospitals were defined as a PTC if they were designated a PTC for paediatric or TYA patients [3, 5]. Some of the PTCs may not have been operating as a PTC over the entire study period, however if a PTC started to operate during the study period the centres would have had a high level of clinical expertise for children and young people and therefore we assigned centres as PTCs based on current practice regardless of when the centre started operating.

In the Yorkshire region PTCs for children and TYA are in Leeds and Sheffield; based on the study population, in Leeds a minimum of 78 children and 57 TYA were treated each year at the PTC and in Sheffield a minimum of 35 children and 18 TYA were treated at the PTC each year. The majority of our study population were treated within the Yorkshire region at these PTCs, however, patients may have been treated at other PTCs throughout the UK, and these hospitals are listed in Additional file 1: Table S1. Patients may also have received treatment at other non-PTC hospitals in Yorkshire or elsewhere in the UK and a total of 89 non-PTC hospitals were included in the analysis with the average volume of patients ranging from less than 1 patient per year to 10 patients per year. An indicator was created to identify if all treating hospitals for each patient were PTC, if some treating hospitals were PTCs or if no treating hospitals were PTCs. The ‘some’ treatment at PTC group included patients who had a mix of treatments at PTC and non-PTC hospitals.

Stage at diagnosis was available for selected diagnostic groups. White cell count was used as a proxy for stage for leukaemia; lymphoma stage was assessed using the Ann Arbor staging system; the Royal Marsden or TNM stage was used for testicular germ cell tumours and FIGO stage for ovarian germ cell tumours. We were unable to obtain sufficient stage information for bone tumours and soft tissue sarcomas. CNS tumours were categorised according to WHO grade. For each main diagnostic group further subgroups according to the ICCC-3 were extracted (see Additional file 2: Tables S2 and Additional file﻿ 3: Tables S3). For bone tumours additional information on the primary site was extracted based on topography and coded as leg, arm, pelvis or ‘other’.

For each patient, treatment was recorded as three separate binary indicators in terms of receiving chemotherapy, surgery or radiotherapy. A combined treatment modality variable was created within each diagnostic group. Treatment modalities with fewer than 30 patients per group were grouped into an ‘Other’ treatment group, whilst patients with no treatment recoded were included as a separate category.

Other data items extracted included age at diagnosis, year of diagnosis, sex, and relapse information. Ethnicity was defined as South Asian or non-South Asian based on the results from a name recognition software program Onomap [10, 11] and individual patient record linkage to hospital episode statistics data as described in previous studies [8, 12]. The area based Townsend deprivation index [13] was obtained from the patient’s postcode at diagnosis.

### Statistical analysis

The percentage of patients treated at PTC by patient characteristics and diagnostic group were calculated. Kaplan Meier plots were used to describe overall survival by level of treatment at PTC. Cox proportional hazards models were used to model survival trends to assess the association between level of treatment at PTC and risk of death. Unadjusted and adjusted models were fitted separately for each diagnostic group. The adjusted model included diagnostic subgroup, age at diagnosis, stage or grade (for leukaemia, lymphoma, CNS tumours and germ cell tumours), treatment, relapse status, sex, year of diagnosis, ethnicity, and deprivation. Models for bone tumours also included primary site in the adjusted model. For bone tumours and soft tissue sarcomas the number of patients receiving some or no treatment at PTC were small, therefore these categories were combined. Predicted survival curves for selected groups of patients were estimated from the adjusted Cox model.

Overall 2% of PTC information was missing. Levels of missing data for stage and grade varied by diagnostic group: white cell count was missing for 18% of cases, lymphoma stage missing for 46%, CNS grade missing for 55% and germ cell tumour stage for 43%. For CNS tumours with missing data, grade was assigned as low grade (WHO Grade I and II) or high grade (WHO grade III and IV) based on tumour morphology [14]. Not all cases could be assigned a grade due to insufficient information, however using this method we were able to assign a grade to another 46% of all CNS tumours, reducing missing grade status to 9%.

Missing PTC status, stage and grade were imputed using multiple imputation models with chained equations [15] and implemented in Stata 14 using the ‘mi’ commands [16]. Each imputation model included all covariates listed previously as well as the Nelson-Aalen estimate for the cumulative hazard function and a death indicator [17].

## Results

In total 2487 patients aged 0–24 years were included in this study: leukaemia (*n* = 684), lymphoma (*n* = 558), CNS tumours (*n* = 547), germ cell tumours (*n* = 364), soft tissue sarcomas (*n* = 171) and bone tumours (n = 163). The total number of treating hospitals for each individual ranged from 1 to 8 (Interquartile range (1 to 2)). Overall 72% of patients received all their treatment at a PTC, 14% of patients had some treatment at a PTC (ranging from 20 to 85%) and 13% of patients received no treatment at a PTC.

The percentage of patients treated at PTC differed by diagnostic group: between 83 and 87% of patients with leukaemia, CNS and bone tumours received all treatment at PTC, compared to 73% for soft tissue sarcomas, 59% for lymphoma patients and 40% for germ cell tumours (Table 1). Age was also significantly associated with level of treatment at PTC: children were more likely to receive all treatment at PTC, with the percentage decreasing with increasing age. Females were more likely to receive all their care at PTC; this was mainly explained by differences for germ cell tumours. The percentage of patients who received all treatment at PTC increased from 70% for patients diagnosed between 1998 and 2005 to 75% for those diagnosed between 2006 and 2009 (Table 1).

**Table 1:** Number and percentage of patients by level of treatment at PTC by patient characteristics

		Level of treatment at PTC				
		All	Some	None	Not known	
Characteristic	Total	n (%)	n (%)	n (%)	n (%)	*p*-value
Diagnostic group						
Leukaemia	684	567 (82.9)	63 (9.2)	48 (7.0)	6 (0.9)	<0.001
Lymphoma	558	328 (58.8)	92 (16.5)	134 (24.0)	4 (0.7)	
CNS tumours	547	478 (87.4)	21 (3.8)	36 (6.6)	12 (2.2)	
Germ cell tumours	364	146 (40.1)	134 (36.8)	82 (22.5)	2 (0.5)	
Soft tissue sarcomas	171	125 (73.1)	29 (17.0)	15 (8.8)	2 (1.2)	
Bone tumours	163	136 (83.4)	17 (10.4)	3 (1.8)	7 (4.3)	
Age group						
0–4 years	496	460 (92.7)	22 (4.4)	6 (1.2)	8 (1.6)	<0.001
5–9 years	371	355 (95.7)	11 (3.0)	2 (0.5)	3 (0.8)	
10–14 years	405	371 (91.6)	18 (4.4)	11 (2.7)	5 (1.2)	
15–19 years	559	329 (58.9)	115 (20.6)	105 (18.8)	11 (2.0)	
20–24 years	655	265 (40.5)	190 (29.0)	194 (29.6)	6 (0.9)	
Sex						
Males	1538	1055 (68.6)	251 (16.3)	208 (13.5)	24 (1.6)	<0.001
Females	949	725 (76.4)	105 (11.1)	110 (11.6)	9 (0.9)	
Diagnosis period						
1998–2005	1671	1168 (69.9)	254 (15.2)	226 (13.5)	23 (1.4)	0.07
2006–2009	816	612 (75.0)	102 (12.5)	92 (11.3)	10 (1.2)	
Total	2487	1780 (71.6)	356 (14.3)	318 (12.8)	33 (1.3)	

Footnote: Row percentages, p-value from Chi squared test for difference between groups

Level of treatment at PTC “Some” ranges from 20% to 85% of all treatments received at PTC

Abbreviations: PTC = Principal Treatment Centre, CNS = Central nervous system

**Fig. 1:**
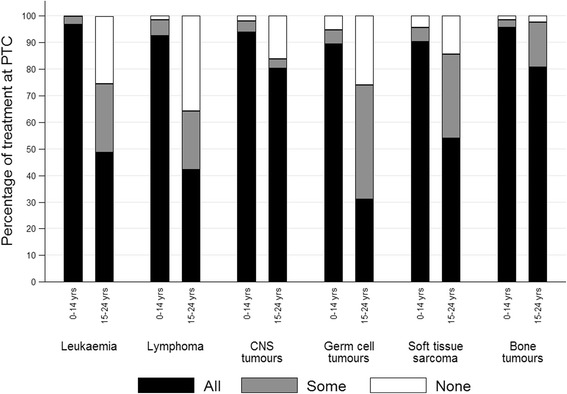
Level of treatment at PTC by diagnostic group and age group, Yorkshire 1998–2009. Footnote: All = patient received all treatment at PTC. Some = patient received some treatment at PTC. Level of treatment at PTC “Some” ranges from 20 to 85% of all treatments received at PTC. None = patient received no treatment at PTC. PTC = Principal Treatment Centre

**Fig. 2:**
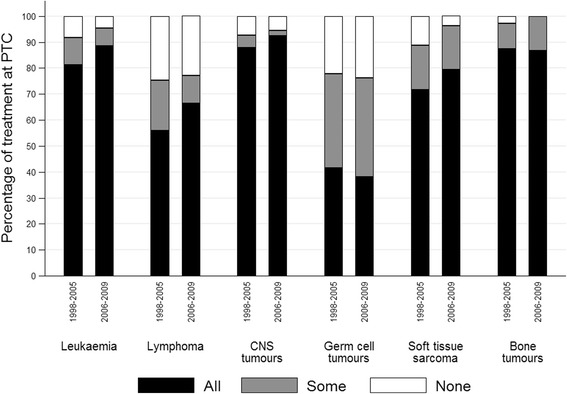
Level of treatment at PTC by diagnostic group and period of diagnosis, Yorkshire 1998–2009. Footnote: All = patient received all treatment at PTC. Some = patient received some treatment at PTC. Level of treatment at PTC “Some” ranges from 20 to 85% of all treatments received at PTC. None = patient received no treatment at PTC. PTC = Principal Treatment Centre

For all diagnostic groups the percentage of children receiving all treatments at PTC was greater than TYA (Fig. 1). For haematological malignancies nearly all children (97% for leukaemia and 93% for lymphoma) had all treatments at PTC compared to around 42–49% of TYA. Approximately 80% of TYA patients with CNS tumours and bone tumours received all treatments at PTC, while this figure was 54% for soft tissue sarcomas and 31% for germ cell tumours. The percentage of patients receiving all treatment at PTC increased significantly over time for patients with leukaemia from 81% for those diagnosed 1998–2005 to 88% for those diagnosed 2006–2009, and for patients with lymphoma from 56 to 66% (Fig. 2).

Leukaemia patients who received some or none of their treatment at a PTC had a significantly increased risk of death in unadjusted analyses (Table 2). After adjustment for patient case-mix a 73% increased risk of death remained for those who received no treatment at PTC with borderline statistical significance; adjusted hazard ratio (HR) =1.73, (95%Confidence Interval (CI) (0.98, 3.04)). Individuals with soft tissue sarcomas who received some or no treatment at a PTC had a reduced risk of death compared to those who received all their treatment at a PTC, an effect which remained significant after our limited adjustment for case-mix; adjusted HR = 0.48 (95%CI (0.23, 0.99)). For lymphoma, CNS tumours, germ cell tumours and bone tumours there were no statistically significant differences in survival between those receiving different levels of treatment at PTC (Table 2, Additional file 4: Figure S1). Similar patterns of results were found when restricted to TYA only (Additional file 5: Table S4).

**Table 2:** Five-year survival (%) and hazard ratios (HR) by level treatment at PTC and diagnostic group

		5 year survival		Unadjusted		Adjusted^a^	
Diagnostic group	Level of treatment at PTC	% Survival	95% CI	HR	95%CI	HR	95%CI
Leukaemia	All	81	(78, 84)	1	-	1	-
	Some	57	(44, 68)	2.34	(1.54, 3.56)	1.61	(0.97, 2.67)
	None	56	(41, 69)	2.66	(1.68, 4.20)	1.73	(0.98, 3.04)
Lymphoma	All	89	(85, 92)	1	-	1	-
	Some	84	(74, 90)	1.45	(0.84, 2.51)	1.51	(0.78, 2.92)
	None	89	(82, 93)	0.98	(0.56, 1.70)	1.12	(0.54, 2.35)
CNS tumours	All	71	(67, 75)	1	-	1	-
	Some	76	(52, 89)	0.6	(0.25, 1.48)	0.48	(0.19, 1.20)
	None	72	(55, 84)	0.98	(0.54, 1.75)	0.66	(0.34, 1.29)
Germ cell tumours	All	91	(85, 95)	1	-	1	-
	Some	94	(88, 97)	0.76	(0.34, 1.70)	1.24	(0.42, 3.65)
	None	95	(88, 98)	0.62	(0.22, 1.17)	1.83	(0.50, 6.76)
Soft tissue sarcomas	All	54	(45, 63)	1	-	1	-
	Some & None	75	(59, 85)	0.44	(0.23, 0.83)	0.48	(0.23, 0.99)
Bone tumours	All	56	(47, 64)	1	-	1	-
	Some & None	60	(36, 78)	0.98	(0.49, 1.97)	0.91	(0.43, 1.88)

Footnote: ^a^Adjusted models from multiple imputation models. Models adjusted for the following

Leukaemia – Diagnostic subgroup, white cell count, age, treatment, relapse, sex, diagnosis year, ethnicity and Townsend area deprivation

Lymphoma – Diagnostic subgroup, stage, age, treatment, relapse, sex, diagnosis year, ethnicity and Townsend area deprivation

CNS tumours – Diagnostic subgroup, grade, age, treatment, relapse, sex, diagnosis year, ethnicity and Townsend area deprivation

Germ cell tumours – Diagnostic subgroup, stage, age, relapse, sex, diagnosis year, ethnicity and Townsend area deprivation (not adjusted for treatment due to collinearity)

Soft tissue sarcomas – Diagnostic subgroup, age, treatment, relapse, sex, diagnosis year, ethnicity and Townsend area deprivation

Bone tumours – Diagnostic subgroup, primary site, age, treatment, relapse, sex, diagnosis year, ethnicity and Townsend area deprivation

Level of treatment at PTC “Some” ranges from 20% to 85% of all treatments received at PTC

Abbreviations: PTC = Principal Treatment Centre, HR = hazard ratio, CI = confidence interval, CNS = Central nervous system

**Fig. 3:**
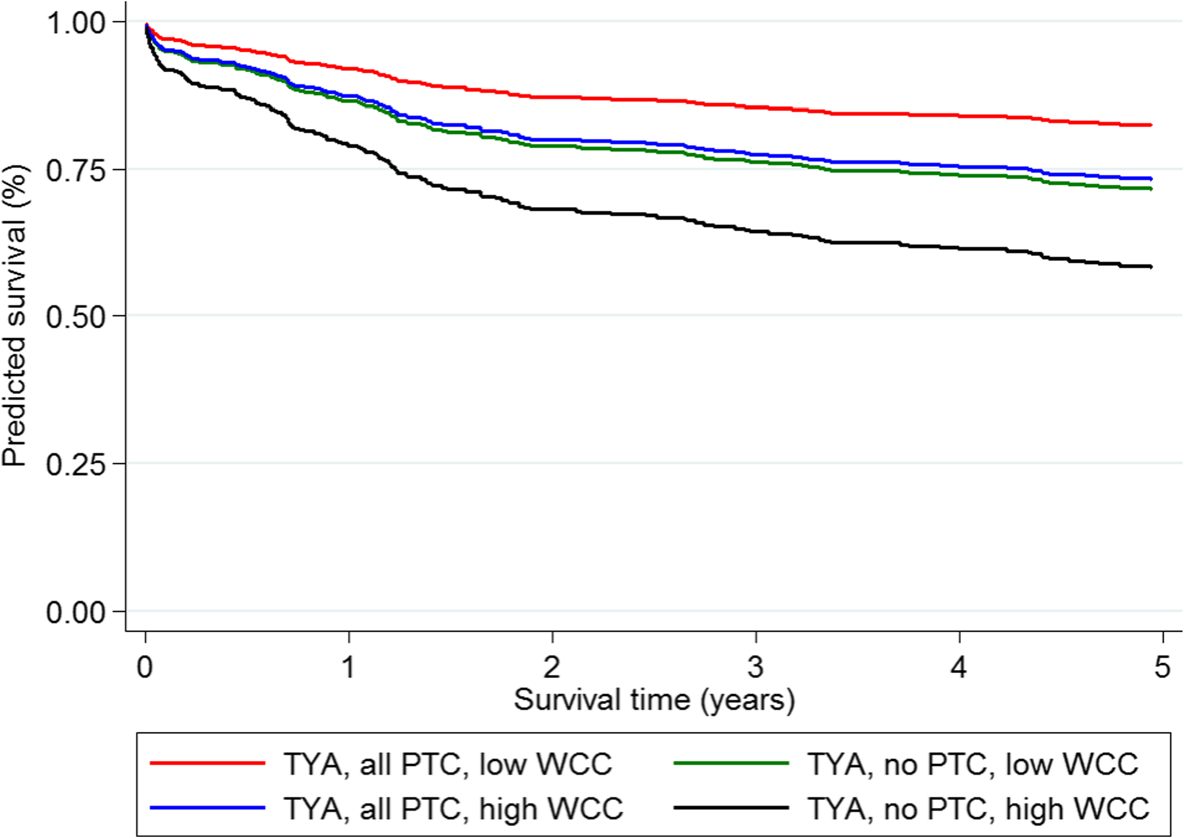
Predicted survival for TYA leukaemia patients with different risk and treatment patterns. Footnote: Comparison of predicted survival for patients with low risk disease (25th percentile value for white cell count) and those with high risk disease (75th percentile value for white cell count) who received either all treatment at PTC or no treatment at PTC. Abbreviations WCC = white cell count, PTC = Principal Treatment Centre, TYA = teenagers and young adults, all PTC = patient received all treatment at PTC, no PTC = patient received no treatment at PTC

Figure 3 shows the predicted survival curves for TYA leukaemia patients, comparing those who received all treatment and no treatment at PTC and with low (equal to the 25th percentile) and high (equal to the 75th percentile) white cell counts to reflect differences in high and low risk cases. Survival outcomes were similar for the high risk cases who received all treatment at PTC to the low risk patients who received no treatment at PTC.

Other variables included in our survival analyses support previously demonstrated findings, indicating the generalisability of our findings. For diagnostic groups where severity of disease was available (leukaemia, lymphoma, CNS tumours and germ cell tumours) the risk of death increased with increasing stage or grade (Additional file 6: Table S5). TYA had an increased risk of death compared to children for leukaemia, CNS tumours, soft tissue sarcomas and germ cell tumours (Additional file 6: Table S5 and Additional file 7: Table S6). For all diagnostic groups there were survival differences by diagnostic subgroup and an increased risk of death for those where no treatment was recorded and those who relapsed. Over the study period the risk of death decreased by 7–10% per year on average for lymphoma and CNS tumours. Increasing deprivation was significantly associated with an increased risk of death for germ cell tumours only, South Asians had an increased risk of death for lymphoma, and there were no significant differences in survival by sex.

## Discussion

This study is one of the first studies to evaluate differences in survival by PCT for children and young people with cancer since the publication of national policy. We were able to assess survival outcomes by provision of care at PTC controlling for patient case-mix. In addition this is one of the first studies to assess the impact of PTC on survival outcomes in TYA in the UK.

We found that access to PTC varied by diagnostic group and age: patients with leukaemia, CNS and bone tumours were more likely to receive all treatment at PTC as were young children compared to TYA aged 15–24 years. Patients with germ cell tumours were less likely to receive treatment at PTC (40% received all treatments at PTC), with clear differences by gender reflecting different types of tumours and treatment patterns for these patients [18]. Males with testicular germ cell tumours may receive surgery at a local hospital and only some will go on to receive chemotherapy, which is likely to be given at PTC, while others will be assessed and put on surveillance at PTC; however we were unable to assess this from our data.

Between 1998 and 2002 in South East England 87% of 10–14 year olds, 55% of 15–19 year olds and 32% of 20–24 year olds were treated at a either a paediatric cancer centre or teenage cancer unit [19]. Details were not available by diagnostic group and findings were based on first treating hospital only. This compared with our data indicating 92% of 10–14 year olds, 59% of 15–19 year olds and 41% of 20–24 year olds received all treatment at PTC in our study, which included patients diagnosed after implementation of the IOG. National data for England for diagnoses between 2001 and 2006 found that only 33% of 15–24 year olds received treatment at a TYA age-specialist centre and this was highest for bone tumours [7]. We also found that for TYA, patients with bone and CNS tumours had the highest proportions receiving all treatment at PTC.

Significant differences in survival outcomes were only evident for leukaemia and soft tissue sarcomas although with differing directions of association. For all other diagnostic groups we did not find an association between place of treatment and survival.

Leukaemia patients receiving all treatment at PTC had better outcomes than those who had some or no treatment at PTC. This difference was only partially explained by patient case-mix. For TYA, predicted survival outcomes were similar for high risk patients receiving all treatment at PTC and low risk patients receiving no treatment at a PTC suggesting that PTC were achieving more favourable outcomes for patients with leukaemia once risk was measured. For childhood leukaemia some studies have shown that treatment at a high volume centre or specialised centre improves survival outcomes [4, 20–23] while some have shown no differences in survival by place of care, [24] including one study focusing on 15–29 year olds with acute leukaemia [25]. Many of these studies included patients diagnosed many years before recent UK policy regarding specialisation of services for this age group.

Soft tissue sarcomas patients who received some or none of their treatment at a PTC had better survival outcomes than those who had all treatment at PTC after some adjustment for patient case-mix. The main caveat associated with this finding is that we were not able to adjust for stage due to lack of information on this variable. We did however adjust for some other case-mix factors thus helping to mediate this effect.

Survival from soft tissue sarcoma is one of the lowest of all diagnostic groups of cancers in children [26] and TYA [27] and outcomes are worse for TYA compared to children [28, 29]. Specialisation of surgical care for soft tissue sarcomas has improved outcomes for adult patients [30, 31], however little is known about the outcomes for children and TYA. Rhabdomyosarcoma patients, aged 0–14 years, diagnosed between 1977 and 1984 and treated at Paediatric Oncology Centres had better survival outcomes than those treated elsewhere in Great Britain, however, this study included children diagnosed many years ago and only adjusted for age and year of diagnosis [20]. We found survival was poorer for patients treated wholly at PTC, most likely due to differences in case-mix with more complex cases with worse prognosis more likely to be referred to PTC. Patients not treated at PTC are more likely to have small tumours completely resected by local surgeons following a presentation where a malignant diagnosis was unexpected. The group of patients receiving some or no treatment at PTC was small and these groups had to be combined in our analysis to retain statistical power. Replication of these findings in other large studies both regionally and nationally is needed, along with further analysis by histological subgroup and collection of stage information to allow for full patient case-mix adjustment.

For paediatric cancers specialised models of care are already well developed in many countries. The development of TYA specialist cancer care is beginning to appear in many European countries [32]. In addition to examining survival outcomes it is also important to quantify differences in the quality of care and patient experience. TYA with cancer are a distinct group with unique needs, both in terms of the cancers diagnosed in this age group and their psychosocial and developmental issues faced. In the UK the BRIGHTLIGHT cohort study has been funded by the National Institute for Health Research to investigate the added value of specialist care for TYA [33] with results shortly to be published.

### Strengths and limitations

The main strengths of this study were the use of high quality data including detailed information on patient case-mix, treatment and stage. We also used information on all hospitals where patients were treated. Other studies have used the first treating hospital only and this does not capture the full patient journey, for example patients who might have initially been treated in a non-specialist centre but then due to complications were referred to a specialist unit.

Limitations were that we did not consider other models of care such as shared care, where patients are managed and discussed at PTC multi-disciplinary team meetings and subsequently treated at local hospitals [2], perhaps closer to the patients home, as we did not have this information. Variations in access to PTC may be due to a lack of appropriate referrals or patient choice and we were unable to assess this in our study.

## Conclusion

Our analysis provides evidence that treatment at PTC improve outcomes for leukaemia patients especially those with high risk disease. There is variation in access to PTC for children and young people with cancer, particularly for TYA. For four of the six diagnostic groups included in our study there were no differences in survival outcomes by the amount of treatment at PTC. However, to fully assess outcomes for soft tissue sarcoma patients further work is needed to ensure appropriate case-mix adjustment and replication of these findings at a national level.

## Additional files

Additional file 1:
**Table S1.** Hospitals with Principal Treatment Centres for children and young people attended by patients diagnosed in Yorkshire 1998–2009 aged 0–24 years (DOCX 19 kb)
Additional file 2:
**Table S2.** Patient case-mix by diagnostic subgroup for leukaemia, lymphoma, CNS tumours and germ cell tumours (DOCX 23 kb)
Additional file 3:
**Table S3.** Patient case-mix by diagnostic subgroup for bone tumours and soft tissue sarcomas(DOCX 19.5 kb)
Additional file 4:
**Figure S1.** Kaplan Meier survival plots by level of treatment at PTC and diagnostic group (TIF 1737 kb)
Additional file 5:
**Table S4.** Hazard ratios for multivariable Cox regression models by level of treatment at PTC and diagnostic group for TYA only (DOCX 25 kb)
Additional file 6:
**Table S5.** Hazard ratios (HR) for patient case-mix variables for leukaemia, lymphoma, CNS tumours and germ cell tumours (DOCX 31.9 kb)
Additional file 7:
**Table S6.** Hazard ratios (HR) for patient case-mix variables for bone tumours and soft tissue sarcomas (DOCX 28.8 kb)

## Abbreviations

CI: Confidence interval
CNS: Central nervous system
HR: Hazard ratio
ICCC-3: International Classification of Childhood Cancer 3rd Edition
IOG: Improving Outcomes Guidance
PTC: Principle Treatment Centre
TYA: Teenagers and young adults
WCC: White cell count
YSRCCYP: Yorkshire Specialist Register of Cancer in Children and Young People
