# Supplementary material for: Access to principal treatment centres and survival rates for children and young people with cancer in Yorkshire, UK
Source: BMC Cancer. 2017 Mar 4;17:168. doi: 10.1186/s12885-017-3160-5 (PMC5336656; doi:10.1186/s12885-017-3160-5)
Supplement: Additional file 1: — Table S1. Hospitals with Principal Treatment Centres for children and young people attended by patients diagnosed in Yorkshire 1998–2009 aged 0–24 years (DOCX 19 kb) [file 12885_2017_3160_MOESM1_ESM.docx]

**Additional file 1**

**Table S1:** Hospitals with Principal Treatment Centres for children and young people attended by patients diagnosed in Yorkshire 1998-2009 aged 0-24 years

| **Hospital** | **City** |
| --- | --- |
| Queen Elizabeth Hospital | Birmingham |
| Royal Orthopaedic Hospital | Birmingham |
| Bristol Children’s Hospital | Bristol |
| Addenbrookes Hospital | Cambridge |
| Leeds General Infirmary | Leeds |
| St James’s Hospital | Leeds |
| Leicester Royal Infirmary | Leicester |
| Great Ormond Street Hospital | London |
| Royal Marsden Hospital | London |
| University College Hospital | London |
| Christie Hospital | Manchester |
| Freeman Hospital | Newcastle |
| Royal Victoria Infirmary | Newcastle |
| Queens Medical Centre | Nottingham |
| John Radcliffe Hospital | Oxford |
| Royal Hallamshire Hospital | Sheffield |
| Sheffield Children’s Hospital | Sheffield |
| Weston Park Hospital | Sheffield |
| Southampton General Hospital | Southampton |
| Clatterbridge Hospital | The Wirral |
