# Supplementary material for: Access to principal treatment centres and survival rates for children and young people with cancer in Yorkshire, UK
Source: BMC Cancer. 2017 Mar 4;17:168. doi: 10.1186/s12885-017-3160-5 (PMC5336656; doi:10.1186/s12885-017-3160-5)
Supplement: Additional file 2: — Table S2. Patient case-mix by diagnostic subgroup for leukaemia, lymphoma, CNS tumours and germ cell tumours (DOCX 23 kb) [file 12885_2017_3160_MOESM2_ESM.docx]

**Additional file 2**

**Table S2:** Patient case-mix by diagnostic subgroup for leukaemia, lymphoma, CNS tumours and germ cell tumours

|  | **Leukaemia (n=684)** | | | **Lymphoma (n=558)** | | | **CNS tumours (n=547)** | | | **Germ cell tumours (n=364)** | | |
| --- | --- | --- | --- | --- | --- | --- | --- | --- | --- | --- | --- | --- |
| **Variable** | **Category** | **n** | **%** | **Category** | **n** | **%** | **Category** | **n** | **%** | **Category** | **n** | **%** |
| **Diagnostic subgroup** | ALL | 490 | (71.6) | HL | 360 | (64.5) | Ependymoma | 56 | (10.2) | Intracranial GCT | 32 | (8.8) |
|  | AML | 150 | (21.9) | NHL | 198 | (35.5) | Astrocytoma | 252 | (46.1) | Malignant extracranial and extragonadal | 29 | (8.0) |
|  | Other | 44 | (6.4) |  |  |  | Embryonal | 92 | (16.8) | Malignant gonadal | 294 | (80.8) |
|  |  |  |  |  |  |  | Other glioma | 60 | (11.0) | Other | 9 | (2.5) |
|  |  |  |  |  |  |  | Other | 87 | (15.9) |  |  |  |
| **Age group** | 0-14 years | 497 | (72.7) | 0-14 years | 189 | (33.9) | 0-14 years | 366 | (66.9) | 0-14 years | 57 | (15.7) |
|  | 15-24 years | 187 | (27.3) | 15-24 years | 369 | (66.1) | 15-24 years | 181 | (33.1) | 15-24 years | 307 | (84.3) |
| **Stage** | WCC<50,000μ/l | 431 | (63.0) | I | 42 | (7.5) | Low grade (I/II) | 298 | (54.5) | I | 126 | (34.6) |
|  | WCC ≥50,000 | 150 | (21.9) | II | 150 | (26.9) | High grade (III/IV) | 196 | (35.8) | II | 45 | (12.4) |
|  | Missing | 103 | (15.1) | III | 70 | (12.5) | Missing | 53 | (9.7) | III | 10 | (2.7) |
|  |  |  |  | IV | 48 | (8.6) |  |  |  | IV | 15 | (4.1) |
|  |  |  |  | Missing | 248 | (44.4) |  |  |  | Missing | 168 | (46.2) |
| **Treatment** | Chemo alone | 550 | (80.4) | Chemo alone | 365 | (65.4) | Surgery alone | 210 | (38.4) | Surgery and chemo | 193 | (53.0) |
|  | Chemo and RT | 66 | (9.6) | Chemo and RT | 75 | (13.4) | Surgery, RT, chemo | 81 | (14.8) | Surgery alone | 90 | (24.7) |
|  | Other | 34 | (5.0) | Chemo and Surgery | 52 | (9.3) | Surgery and chemo | 59 | (10.8) | Surgery and RT | 37 | (10.2) |
|  | No treatment recorded | 34 | (5.0) | Other | 39 | (7.0) | Other | 121 | (22.1) | Other | 33 | (9.1) |
|  |  |  |  | No treatment recorded | 27 | (4.8) | No treatment recorded | 76 | (13.9) | No treatment recorded | 11 | (3.0) |
| **Relapsed** | No | 571 | (83.5) | No | 482 | (86.4) | No | 465 | (85.0) | No | 341 | (93.7) |
|  | Yes | 113 | (16.5) | Yes | 76 | (13.6) | Yes | 82 | (15.0) | Yes | 23 | (6.3) |
| **Sex** | Male | 388 | (56.7) | Male | 336 | (60.2) | Male | 296 | (54.1) | Male | 316 | (86.8) |
|  | Female | 296 | (43.3) | Female | 222 | (39.8) | Female | 251 | (45.6) | Female | 48 | (13.2) |
| **Diagnosis period** | 1998-2005 | 463 | (67.7) | 1998-2005 | 382 | (68.5) | 1998-2001 | 363 | (66.4) | 1998-2001 | 236 | (64.8) |
|  | 2006-2009 | 221 | (32.3) | 2006-2009 | 176 | (31.5) | 2006-2009 | 184 | 33.6) | 2006-2009 | 128 | (35.2) |
| **Ethnicity** | Non-South Asian | 623 | (91.1) | Non-South Asian | 484 | (86.7) | Non-South Asian | 509 | (93.0) | Non-South Asian | 338 | (92.9) |
|  | South Asian | 61 | (8.9) | South Asian | 74 | (13.3) | South Asian | 38 | (7.0) | South Asian | 26 | (7.1) |

Abbreviations: CNS = Central nervous system, ALL = Acute lymphoblastic leukaemia, AML = Acute myeloid leukaemia, HL = Hodgkin lymphoma, NHL = Non-Hodgkin lymphoma, GCT = Germ cell tumour, WCC = white cell count, RT = radiotherapy
