# Supplementary material for: Access to principal treatment centres and survival rates for children and young people with cancer in Yorkshire, UK
Source: BMC Cancer. 2017 Mar 4;17:168. doi: 10.1186/s12885-017-3160-5 (PMC5336656; doi:10.1186/s12885-017-3160-5)
Supplement: Additional file 3: — Table S3. Patient case-mix by diagnostic subgroup for bone tumours and soft tissue sarcomas(DOCX 19.5 kb) [file 12885_2017_3160_MOESM3_ESM.docx]

**Additional file S3**

**Table S3:** Patient case-mix by diagnostic subgroup for bone tumours and soft tissue sarcomas

|  | **Soft tissue sarcomas (n=171)** | | | **Bone tumours (n=163)** | | |
| --- | --- | --- | --- | --- | --- | --- |
| **Variable** | **Category** | **n** | **%** | **Category** | **n** | **%** |
| **Diagnostic subgroup** | Rhabdomyosarcoma | 60 | (35.1) | Osteosarcoma | 81 | (49.7) |
|  | Fibrosarcoma | 19 | (11.1) | Chondrosarcoma | 11 | (6.7) |
|  | Other | 92 | (53.8) | Ewing’s sarcoma | 68 | (41.7) |
|  |  |  |  | Other | 3 | (1.8) |
| **Age group** | 0-14 years | 94 | (55.0) | 0-14 years | 69 | (42.3) |
|  | 15-24 years | 77 | (45.0) | 15-24 years | 94 | (57.7) |
| **Treatment** | Surgery alone | 37 | (21.6) | Chemo alone | 61 | (37.4) |
|  | Chemo alone | 38 | (22.2) | Chemo and surgery | 45 | (27.6) |
|  | Surgery and Chemo | 35 | (20.5) | Other | 41 | (25.2) |
|  | Other | 50 | (29.2) | No treatment recorded | 16 | (9.8) |
|  | No treatment recorded | 11 | (6.4) |  |  |  |
| **Primary site** |  |  |  | Leg | 93 | (57.1) |
|  |  |  |  | Arm | 18 | (11.0) |
|  |  |  |  | Pelvis | 22 | (13.5) |
|  |  |  |  | Other | 30 | (18.4) |
| **Relapsed** | No | 134 | (78.4) | No | 113 | (69.3) |
|  | Yes | 37 | (21.6) | Yes | 50 | (30.7) |
| **Sex** | Male | 100 | (58.5) | Male | 102 | (62.6) |
|  | Female | 71 | (41.5) | Female | 61 | (37.4) |
| **Diagnosis period** | 1998-2005 | 118 | (69.0) | 1998-2005 | 109 | (66.9) |
|  | 2006-2009 | 53 | (31.0) | 2006-2009 | 54 | (33.1) |
| **Ethnicity** | Non-South Asian | 157 | (91.8) | Non-South Asian | 147 | (90.2) |
|  | South Asian | 14 | (8.2) | South Asian | 16 | (9.8) |
