# Supplementary figures and images for: Access to principal treatment centres and survival rates for children and young people with cancer in Yorkshire, UK
Source: BMC Cancer. 2017 Mar 4;17:168. doi: 10.1186/s12885-017-3160-5 (PMC5336656; doi:10.1186/s12885-017-3160-5)

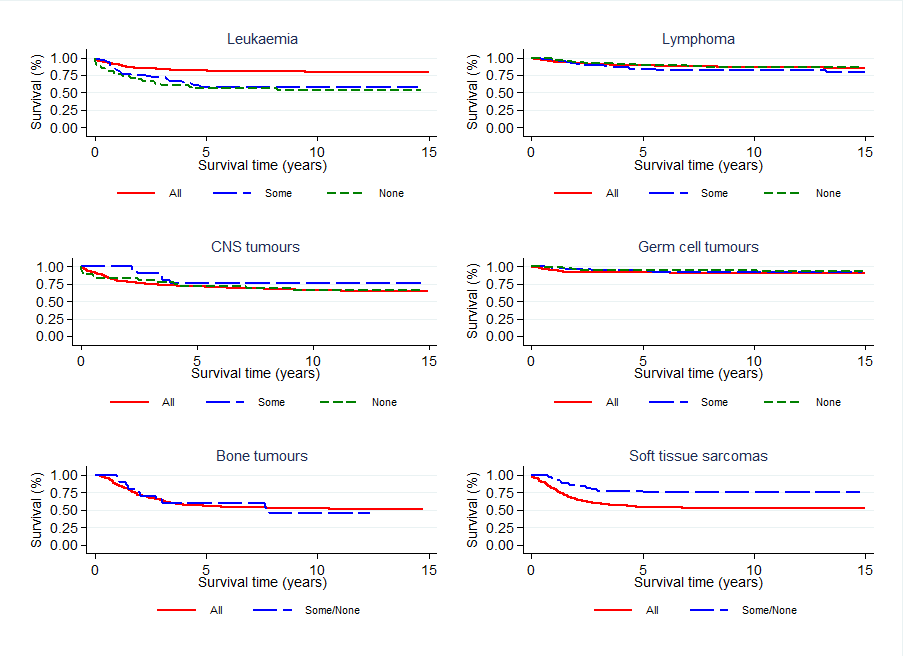

Supplement: Additional file 4: — Figure S1. Kaplan Meier survival plots by level of treatment at PTC and diagnostic group (TIF 1737 kb) [file 12885_2017_3160_MOESM4_ESM.tif]
