# Supplementary material for: Access to principal treatment centres and survival rates for children and young people with cancer in Yorkshire, UK
Source: BMC Cancer. 2017 Mar 4;17:168. doi: 10.1186/s12885-017-3160-5 (PMC5336656; doi:10.1186/s12885-017-3160-5)
Supplement: Additional file 5: — Table S4. Hazard ratios for multivariable Cox regression models by level of treatment at PTC and diagnostic group for TYA only (DOCX 25 kb) [file 12885_2017_3160_MOESM5_ESM.docx]

**Additional file 5**

**Table S4:** Hazard ratios for multivariable Cox regression models by level of treatment at PTC and diagnostic group for TYA only

| **Diagnostic group** | **Level of treatment at PTC** | **Adjusted HR** | **95%CI** |
| --- | --- | --- | --- |
| Leukaemia | All | 1 | - |
|  | Some | 1.63 | (0.90, 2.92) |
|  | None | 1.79 | (0.99, 3.25) |
| Lymphoma | All |  |  |
|  | Some | 1.27 | (0.61, 2.65) |
|  | None | 0.99 | (0.47, 2.10) |
| CNS tumours | All |  |  |
|  | Some | 0.55 | (0.13, 2.52) |
|  | None | 0.91 | (0.42, 1.94) |
| Germ cell tumours | All |  |  |
|  | Some | 1.52 | (0.46, 5.03) |
|  | None | 2.56 | (0.46, 14.20) |
| Soft tissue sarcoma | All |  |  |
|  | Some & None | 0.26 | (0.10, 0.66) |
| Bone tumours | All |  |  |
|  | Some & None | 1.14 | (0.52, 2.52) |

Adjusted models from multiple imputation models. Models adjusted for the following:

Leukaemia – Diagnostic subgroup, white cell count, age, treatment, relapse, sex, diagnosis year, ethnicity and Townsend area deprivation

Lymphoma – Diagnostic subgroup, stage, age, treatment, relapse, sex, diagnosis year, ethnicity and Townsend area deprivation

CNS tumours – Diagnostic subgroup, grade, age, treatment, relapse, sex, diagnosis year, ethnicity and Townsend area deprivation

Germ cell tumours – Diagnostic subgroup, stage, age, relapse, sex, diagnosis year, ethnicity and Townsend area deprivation (not adjusted for treatment due to collinearity)

Soft tissue sarcomas – Diagnostic subgroup, age, treatment, relapse, sex, diagnosis year, ethnicity and Townsend area deprivation

Bone tumours – Diagnostic subgroup, primary site, age, treatment, relapse, sex, diagnosis year, ethnicity and Townsend area deprivation

Abbreviations: PTC = Principal Treatment Centre, HR = hazard ratio, CI = confidence interval, CNS = Central nervous system

Level of treatment at PTC “Some” ranges from 20% to 85% of all treatments received at PTC
