# Supplementary material for: Access to principal treatment centres and survival rates for children and young people with cancer in Yorkshire, UK
Source: BMC Cancer. 2017 Mar 4;17:168. doi: 10.1186/s12885-017-3160-5 (PMC5336656; doi:10.1186/s12885-017-3160-5)
Supplement: Additional file 6: — Table S5. Hazard ratios (HR) for patient case-mix variables for leukaemia, lymphoma, CNS tumours and germ cell tumours (DOCX 31.9 kb) [file 12885_2017_3160_MOESM6_ESM.docx]

**Additional file 6**

**Table S5:** Hazard ratios (HR) for patient case-mix variables for leukaemia, lymphoma, CNS tumours and germ cell tumours

|  | **Leukaemia (n=684)** | | | **Lymphoma (n=558)** | | | **CNS tumours (n=547)** | | | **Germ cell tumours (n=364)** | | |
| --- | --- | --- | --- | --- | --- | --- | --- | --- | --- | --- | --- | --- |
| **Variable** | **Category** | **HR** | **95%CI** | **Category** | **HR** | **95%CI** | **Category** | **HR** | **95%CI** | **Category** | **HR** | **95%CI** |
| Diagnostic subgroup | ALL | 1 | - | HL | 1 | - | Ependymoma | 0.4 | (0.21, 0.76) | Malignant gonadal | 1 | - |
|  | AML | 1.58 | (1.10, 2.26) | NHL | 1.97 | (1.16, 3.33) | Astrocytoma | 1 | - | Other | 5.67 | (1.55, 20.7) |
|  | Other | 0.42 | (0.19, 0.95) |  |  |  | Embryonal | 0.58 | (0.37, 0.90) |  |  |  |
|  |  |  |  |  |  |  | Other glioma | 1.34 | (0.82, 2.18) |  |  |  |
|  |  |  |  |  |  |  | Other | 0.47 | (0.25, 0.88) |  |  |  |
| Age group | 0-14 years | 1 | - | 0-14 years | 1 | - | 0-14 years | 1 | - | 0-14 years | 0.24 | (0.05, 1.07) |
|  | 15-24 years | 1.55 | (1.01, 2.38) | 15-24 years | 1.03 | (0.55, 1.93) | 15-24 years | 1.45 | (1.01, 2.08) | 15-24 years | 1 | - |
| Stage | per 20% increase | 1.04 | (1.02, 1.06) | I | 1 | - | Low (I/II) | 1 | - | I | 1 | - |
|  |  |  |  | II | 1.18 | (0.39, 3.62) | High (III/IV) | 6.42 | (4.11, 10.0) | II | 2.15 | (0.50, 9.28) |
|  |  |  |  | III | 2.07 | (0.57, 7.45) |  |  |  | III | 3.59 | (0.38, 33.85) |
|  |  |  |  | IV | 5.65 | (1.53, 20.8) |  |  |  | IV | 15.04 | (3.08, 73.39) |
| Treatment | Chemo alone | 1 | - | Chemo alone | 1 | - | Surgery alone | 1 | - | Surgery and chemo |  |  |
|  | Chemo and RT | 0.94 | (0.58, 1.51) | Chemo and RT | 1.04 | (0.55, 1.97) | Surgery, RT, chemo | 1.81 | (1.05, 3.14) | Surgery alone |  |  |
|  | Other | 1.43 | (0.74, 2.78) | Chemo and Surgery | 0.28 | (0.09, 0.84) | Surgery and chemo | 2.29 | (1.26, 4.17) | Surgery and RT |  |  |
|  | No treatment recorded | 2.83 | (1.64, 4.91) | Other | 1.14 | (0.41, 3.14) | Other | 1.94 | (1.16, 3.24) | Other |  |  |
|  |  |  |  | No treatment recorded | 3.12 | (1.16, 8.36) | No treatment recorded | 2.09 | (1.15, 3.77) | No treatment recorded |  |  |
| Relapse | No | 1 | - | No | 1 | - | No | 1 | - | No | 1 | - |
|  | Yes | 2.82 | (1.99, 4.01) | Yes | 7.81 | (4.64, 13.2) | Yes | 1.7 | (1.18, 2.46) | Yes | 2.77 | (0.78, 9.86) |
| Sex | Male | 1 | - | Male | 1 | - | Male | 1 | - | Male | 1 | - |
|  | Female | 1.19 | (0.87, 1.63) | Female | 0.93 | (0.57, 1.50) | Female | 1.19 | (0.85, 1.65) | Female | 1.06 | (0.28, 4.04) |
| Diagnosis year | Per year | 0.97 | (0.92, 1.02) | Per year | 0.9 | (0.83, 0.97) | Per year | 0.93 | (0.89, 0.98) | Per year | 1.1 | (0.96, 1.25) |
| Ethnicity | Non South Asian | 1 | - | Non South Asian | 1 | - | Non South Asian | 1 | - | Non South Asian | 1 | - |
|  | South Asian | 1.45 | (0.81, 2.61) | South Asian | 2.04 | (1.02, 4.11) | South Asian | 1.17 | (0.65, 2.11) | South Asian | 1.19 | (0.29, 4.85) |
| Townsend | per SD increase | 1.03 | (0.88, 1.22) | per SD increase | 0.88 | (0.66, 1.18) | per SD increase | 1.17 | (0.98, 1.40) | Per SD increase | 1.4 | (0.92, 2.13) |

Models adjusted for all variables in table plus level of treatment at PTC

Abbreviations: CNS = Central nervous system, HR = Hazard ratio, CI = confidence interval, ALL = Acute lymphoblastic leukaemia, AML = Acute myeloid leukaemia, HL = Hodgkin lymphoma, NHL = Non-Hodgkin lymphoma, WCC = white cell count, RT = radiotherapy, SD = Standard deviation
