# Supplementary material for: Access to principal treatment centres and survival rates for children and young people with cancer in Yorkshire, UK
Source: BMC Cancer. 2017 Mar 4;17:168. doi: 10.1186/s12885-017-3160-5 (PMC5336656; doi:10.1186/s12885-017-3160-5)
Supplement: Additional file 7: — Table S6. Hazard ratios (HR) for patient case-mix variables for bone tumours and soft tissue sarcomas (DOCX 28.8 kb) [file 12885_2017_3160_MOESM7_ESM.docx]

**Additional file 7**

**TableS6:** Hazard ratios (HR) for patient case-mix variables for bone tumours and soft tissue sarcomas

|  | | **Soft Tissue Sarcoma (n=171)** | | | **Bone tumours (n=163)** | | |
| --- | --- | --- | --- | --- | --- | --- | --- |
| **Variable** | **Category** | | **HR** | **95%CI** | **Category** | **HR** | **95%CI** |
| Diagnostic subgroup | Rhabdomyosarcoma | | 1 | - | Osteosarcoma | 1 | - |
|  | Fibrosarcoma | | 1.15 | (0.47, 2.77) | Ewing’s sarcoma | 0.75 | (0.42, 1.36) |
|  | Other | | 1.75 | (1.01, 3.04) | Other | 0.27 | (0.07, 1.15) |
| Age group | 0-14 years | | 1 | - | 0-14 years | 1 | - |
|  | 15-24 years | | 2.05 | (1.18, 3.57) | 15-24 years | 0.93 | (0.55, 1.56) |
| Treatment | Surgery alone | | 1 | - | Chemo alone | 1 | - |
|  | Chemo alone | | 20.69 | (4.58, 93) | Chemo and surgery | 0.37 | (0.19, 0.72) |
|  | Surgery and Chemo | | 8.05 | (1.76, 36.8) | Other | 0.55 | (0.30, 1.02) |
|  | Other | | 7.47 | (1.67, 33.5) | No treatment recorded | 1.31 | (0.52, 3.28) |
|  | No treatment recorded | | 14.48 | (2.75, 76) |  |  |  |
| Primary site |  | |  |  | Leg | 1 | - |
|  |  | |  |  | Arm | 1.44 | (0.65, 3.20) |
|  |  | |  |  | Pelvis | 2.57 | (1.22, 5.41) |
|  |  | |  |  | Other | 1.84 | (0.81, 4.16) |
| Relapse | No | | 1 | - | No | 1 | - |
|  | Yes | | 3.1 | (1.81, 5.29) | Yes | 3.19 | (1.93, 5.28) |
| Sex | Male | | 1 | - | Male | 1 | - |
|  | Female | | 0.57 | (0.32, 1.02) | Female | 0.73 | (0.43, 1.24) |
| Diagnosis year | Per year | | 1.03 | (0.96, 1.11) | Per year | 0.97 | (0.90, 1.04) |
| Ethnicity | Non South Asian | | 1 | - | Non South Asian | 1 | - |
|  | South Asian | | 1.21 | (0.32, 4.67) | South Asian | 0.65 | (0.26, 1.61) |
| Townsend | per SD increase | | 0.85 | (0.64, 1.13) | per SD increase | 0.93 | (0.71, 1.22) |

Footnote: Models adjusted for all variables in table plus level of treatment at PTC

Abbreviations: HR = Hazard ratio, CI = confidence interval, SD = Standard deviation
